# Reversible QRS fragmentation in Takotsubo cardiomyopathy

**DOI:** 10.1002/joa3.12790

**Published:** 2022-10-13

**Authors:** Yan‐Kiu Li, Bernard M.‐Y. Cheung, Hung‐Fat Tse, Yap‐Hang Chan

**Affiliations:** ^1^ Department of Medicine, School of Clinical Medicine Queen Mary Hospital, University of Hong Kong Hong Kong Hong Kong

An 80‐year‐old lady with a history of diabetes mellitus presented to the emergency room with palpitations. Electrocardiogram revealed ST‐segment elevation over the inferior and lateral leads. Notably, the QRS complexes across precordial leads V4‐6 and aVR were fragmented. A positive deflection was seen in lead V1, embedded within the QRS complex, which is resemblant to the Epsilon wave morphology (Figure 1, left columns). Despite such findings and raised serum troponin, she was free from chest pain. Transthoracic echocardiography showed left ventricular (LV) apical ballooning with severe hypokinesis, corroborated by invasive left ventriculography. Coronary angiography revealed no occlusive lesion. The diagnosis was Takotsubo cardiomyopathy (TTC). (Figure 2).

**FIGURE 1:**
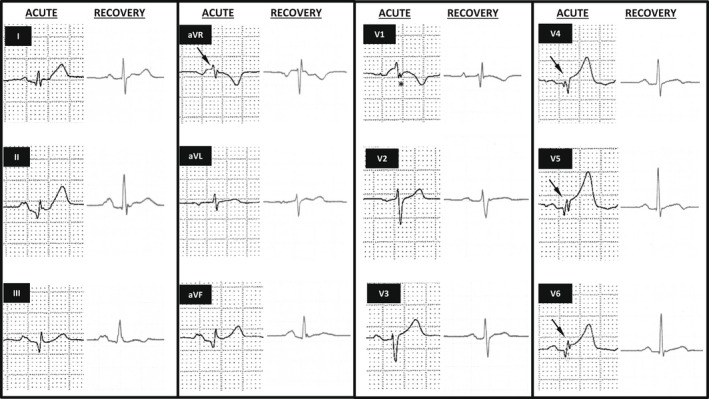
Fragmentation of QRS complexes (annotated in arrows) in leads aVR, V4‐6 and presence of an epsilon wave‐like morphology (*) in lead V1 during the acute phase. These changes were resolved 3 months later.

**FIGURE 2:**
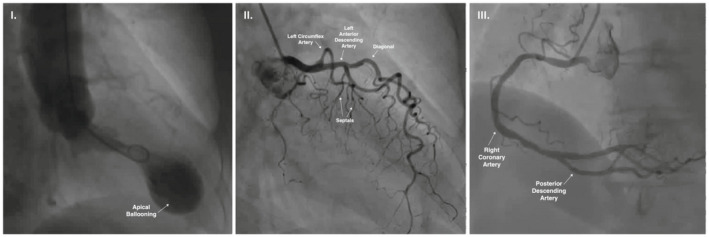
Invasive left ventriculography depicting apical ballooning cardinal of Takotsubo cardiomyopathy. Coronary angiography of the left and right systems revealed no explanatory occlusive lesion.

The finding of fragmented QRS in TTC is of electrophysiological interest. Arrhythmic manifestations complicating TTC affects up to one‐quarter of patients, and half is life‐threatening (13.5%).^1^ Symptomatic atrioventricular block and less commonly sinus nodal dysfunction may mandate cardiac pacing and are due to a secondary increase in vagal tone in sympathetic overdrive. The origin of ventricular tachycardia (VT)/ fibrillation remained controversial in TTC. While QT prolongation is well‐recognized in TTC that provided a substrate for polymorphic VT, the origin of monomorphic VT was believed to be catecholaminergic or automatic, and unlikely reentrant based on classical lack of myocardial fibrosis detectable as late gadolinium MRI enhancement in TCC.^2^, ^3^ However, latest research provided revoking evidence by identifying diffuse fibrosis/ micronecrosis through T1‐mapping in TTC.^4^ QRS fragmentation, a marker of myocardial fibrosis, is predictive of sudden cardiac death in patients with ischemic and non‐ischemic cardiomyopathy. Its presence in the inferior leads, in particular, was identified as an ominous sign in patients with known cardiomyopathy for subsequent sudden death,^5^ while lateral lead predominance conferred excess risks in subjects without structural heart disease. Whether fragmented QRS has a prognostic role in Takotsubo cardiomyopathy remained unclear. Furthermore, myocardial edema during a hyperacute phase in TTC may predispose to transient electrical inhomogeneity.^2^ On the contrary, the Epilson wave, an electrocardiographic finding characteristic of arrhythmogenic right ventricular cardiomyopathy, together with QRS fragmentation are both indicators of delayed ventricular depolarization. While the Epilson wave has been reported in myocarditis, how it is related to myocardial edema in the hyperacute phase of TTC and whether it may have a prognostic role remained obscure.

In our patient, no significant nodal dysfunction or ventricular tachyarrhythmia occurred. She was treated with aspirin (80 mg daily), lisinopril (2.5 mg daily), bisoprolol fumarate (1.25 mg daily), and atorvastatin (20 mg daily). Three months later, reassessment echocardiography showed recovered LV systolic function with an ejection fraction of 65%, and resolution of apical ballooning. These were accompanied by a complete reversal of QRS fragmentation and the disappearance of the Epsilon wave‐like morphology (Figure 1, rights columns). Ambulatory Holter assessment showed nil evidence of electrical instability. This succinct *Spotlight* thus re‐capitulates the fundamental electro‐pathophysiological basis implicated in the natural course of TTC, in which further research is needed.

## FUNDING INFORMATION

Li Shu Fan Medical Fellowship for Internal Medicine 2021/22 (Y.H.C.)

## CONFLICT OF INTERESTS

None declared.
